# Non‐linearity in event runoff generation in a small agricultural catchment

**DOI:** 10.1002/hyp.14667

**Published:** 2022-08-23

**Authors:** Mariette Vreugdenhil, Borbála Széles, José Luis Salinas, Peter Strauß, Markus Oismueller, Patrick Hogan, Wolfgang Wagner, Juraj Parajka, Günter Blöschl

**Affiliations:** ^1^ Research Group Remote Sensing, Department of Geodesy and Geoinformation TU Wien Vienna Austria; ^2^ Centre for Water Resource Systems TU Wien Vienna Austria; ^3^ Institute for Hydrology and Water Resource Management TU Wien Vienna Austria; ^4^ Institute for Land and Water Management Research Federal Agency for Water Management Petzenkirchen Austria

**Keywords:** connectivity, flow paths, groundwater, non‐linearity, precipitation, runoff generation, scaling, seasonality, soil moisture

## Abstract

Understanding the role of soil moisture and other controls in runoff generation is important for predicting runoff across scales. This paper aims to identify the degree of non‐linearity of the relationship between event peak runoff and potential controls for different runoff generation mechanisms in a small agricultural catchment. The study is set in the 66 ha Hydrological Open Air Laboratory, Austria, where discharge was measured at the catchment outlet and for 11 sub‐catchments or hillslopes with different runoff generation mechanisms. Peak runoff of 73 events was related to three potential controls: event precipitation, soil moisture and groundwater levels. The results suggest that the hillslopes dominated by ephemeral overland flow exhibit the most non‐linear runoff generation behaviour for its controls; runoff is only generated above a threshold of 95% of the maximum soil moisture. Runoff generation through tile drains and in wetlands is more linear. The largest winter and spring events at the catchment outlet are caused by runoff from hillslopes with shallow flow paths (ephemeral overland flow and tile drainage mechanisms), while the largest summer events are caused by other hillslopes, those with deeper flow paths or with saturation areas throughout the year. Therefore, the response of the entire catchment is a mix of the various mechanisms, and the groundwater contribution makes the response more linear. The implications for hydrological modelling are discussed.

## Graphical abstract

The non‐linearity of the relationships between event runoff peak and its controls depends on the runoff generation mechanisms: Ephemeral overland flow systems are the most non‐linear and related to soil moisture; tile drainage and wetland systems are less non‐linear; more linear behaviour occurs as the catchment size increases.

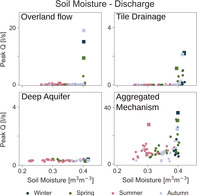

## 1 INTRODUCTION

Soil moisture is frequently found to be the dominant control on runoff processes across scales, especially in humid regions (Meyles et al., 2003; Rodríguez‐Blanco et al., 2012; Western & Grayson, 1998). As the catchment wets up, an increasingly larger area may contribute to event runoff through different runoff generation mechanisms. Locally, infiltration tends to decrease, which increases infiltration excess runoff. As shallow or perched aquifers reach the surface, the size of the saturated areas expands (e.g., Silasari et al., 2017) and an increasing fraction of rainfall is transformed into runoff (Han et al., 2012; Hrnčíř et al., 2010). On the other hand, the connectivity of the locally produced runoff with the stream, both on the surface and in the near sub‐surface, tends to increase, resulting in the activation of preferential flow paths and thus an increase in stream runoff (Chirico et al., 2003; Grayson et al., 1997; Sidle et al., 2000; Votrubova et al., 2017; Western et al., 2001). Due to these reasons, the soil moisture state is generally considered a key control of runoff generation processes (Detty & McGuire, 2010; Penna et al., 2011; Saffarpour et al., 2016; Zehe et al., 2010).

The soil moisture–runoff relationship may exhibit different characteristic forms, depending on the runoff generation processes related to catchment characteristics, rainstorm characteristics, plant management characteristics, climate and scale. Matrix flow switching to preferential flow may induce a very non‐linear threshold relationship at the plot scale (Zehe & Blöschl, 2004). Sidle et al. (1995, 2000) observed a highly non‐linear relationship on both the hillslope and headwater scales on steep forested terrain in Japan. During dry conditions, saturated overland flow from the riparian zone and channel interception contributed to runoff, while during wet conditions, geomorphic hollows started to contribute to stormflow above a saturation threshold and preferential flow pathways expanded (Sidle et al., 1995, 2000). A similar threshold‐like relationship between runoff and soil moisture may be produced by a shallow riparian water table connecting to the stream, as demonstrated by James and Roulet (2009) for eight small, nested forest catchments in Canada. Previous studies (e.g., Chifflard et al., 2018; Detty & McGuire, 2010; Meyles et al., 2003; Penna et al., 2011; Sidle et al., 1995, 2000) reported that below a certain soil moisture threshold, runoff was generated primarily in the near‐stream zone, while the hillslopes started to contribute only above the threshold. Threshold behaviour in hydrology is a particular form of non‐linearity, where a change in processes or switch in regimes occurs (Zehe & Blöschl, 2004), resulting in much more intense hydrologic response than usual.

However, other controls on runoff generation can be equally or even more critical. Event precipitation depth contributes to saturating the soil during an event and thus enhances runoff through the saturation excess mechanism, while rainfall intensity peaks are particularly important in the infiltration excess mechanism (Szilagyi, 2007). Analysis of event runoff coefficients thus suggests that they usually depend both on event rainfall and antecedent soil moisture (Merz & Blöschl, 2009; Norbiato et al., 2009; Rodríguez‐Blanco et al., 2012). Additionally, pipe flow may contribute to non‐linearity. For example, in a comparison of four small catchments located in Japan and in the United States Uchida et al. (2005) found that initiation of pipe flow was threshold‐dependent and controlled by event rainfall and antecedent soil moisture. They also found that the ratio of total pipe flow to total hillslope runoff was similar for all events, pointing towards a functional similarity of other runoff generation processes with pipe flow. Tile drains may operate similarly. For example, Lam et al. (2016) found that runoff response from two tile drains in an agricultural field with sandy loam soil in southern Ontario, Canada, occurred mainly in winter when the soil moisture exceeded a threshold of about 0.5 m^3^/m^3^ in the top 10 cm of the soil. Spence et al. (2007) observed runoff from 50 ha prairie catchment in Canada to be controlled by storage thresholds. Graham and McDonnell (2010) showed that the threshold behaviour of runoff generation in a small forested catchment in New Zealand is due to storm spacing and potential evaporation on the one hand, and bedrock permeability and bedrock topography on the other hand. Ross et al. (2021) observed rainfall depth thresholds in runoff generation for most of the 21 sites analysed in Canada, the United States, Australia and New Zealand, and rainfall intensity thresholds for some sites. They found the threshold behaviour to be sensitive to antecedent soil moisture.

While a wide variety of studies on the soil moisture‐runoff relationship exist, they either focus on a single hillslope or a small catchment where few types of runoff mechanism are present, or compare threshold behaviour across catchments and environments. This makes it difficult to disentangle the contributions of different runoff generation mechanisms under the same environmental conditions. The aim of this paper therefore is to understand the non‐linearity of runoff generation by contrasting different mechanisms in the same catchment. The study is set in the 66 ha Hydrological Open Air Laboratory (HOAL) in Austria, which exhibits a variety of runoff generation mechanisms (e.g., springs, tile drains, overland flow, wetlands) and where runoff, rainfall, soil moisture and groundwater levels are measured at high spatial and temporal resolutions (Blöschl et al., 2016; Exner‐Kittridge et al., 2016). This work thus goes beyond the existing literature by evaluating the behaviour of different runoff generation mechanisms in a comparative way.

## 2 DATA AND METHODS

### 2.1 Study area

**FIGURE 1:**
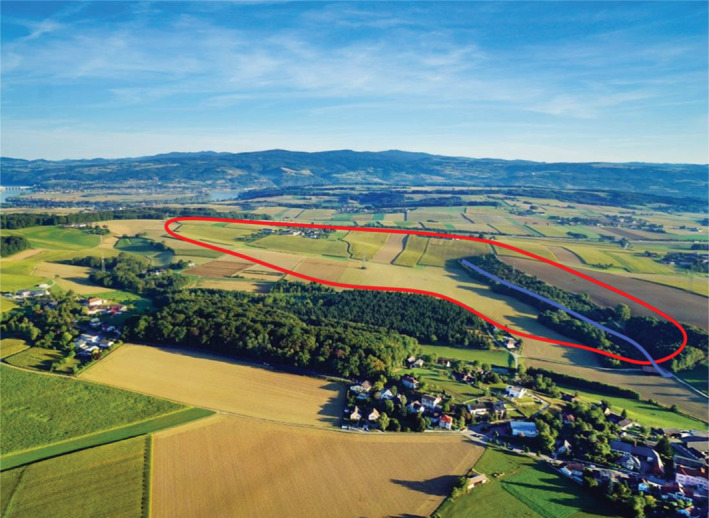
Aerial photograph of the 66 ha Hydrological Open Air Laboratory (HOAL) in lower Austria. Red line indicates the topographic catchment boundary, blue line indicates the main stream of the catchment. Photo: A. Eder

The 66 ha HOAL is located in Petzenkirchen, Austria, about 100 km west of Vienna (Figure 1). The main stream is the Seitengraben, with a mean annual runoff of 4.1 L/s (195 mm/year) at the outlet (1990–2014). Elevations range from 268 to 323 m above sea level with a mean slope of 8%. Soils have medium to poor infiltration capacity due to a relatively high‐clay content between 20% and 30%, and the dominant soil types are Cambisols and Planosols. In Planosols, standing water occurs due to a low‐permeability clay layer 40 cm below the surface. Land use consists of agriculture (87%), forest (6%), pasture (5%) and paved area (2%). The climate is humid with mean annual air temperature and rainfall of 9.5°C and 823 mm/year, respectively (1990–2014). Monthly rainfall and hourly rainfall intensities are highest during summer.

**FIGURE 2:**
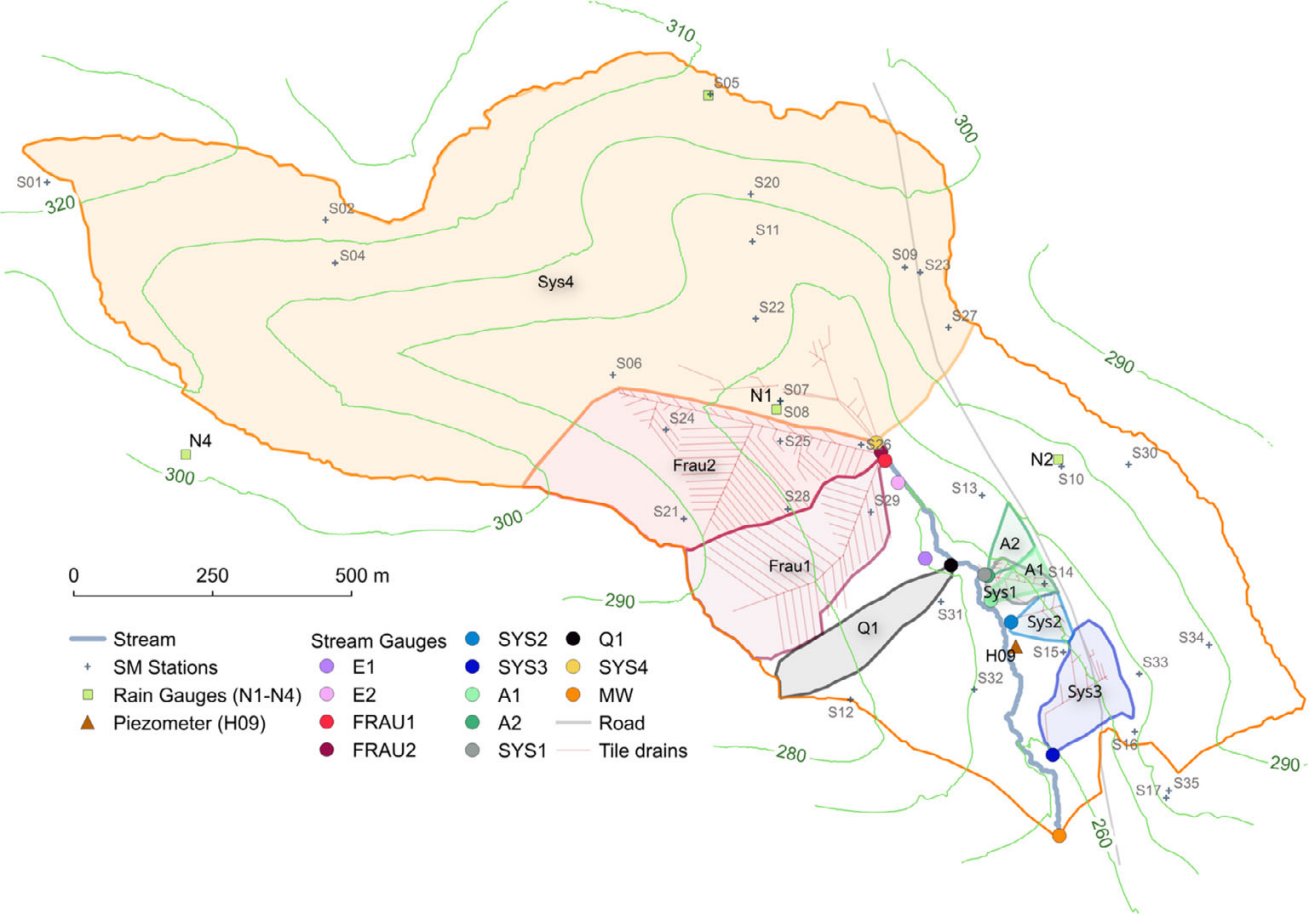
Hydrological Open Air Laboratory (HOAL) showing locations of stream gauges (full circles) with sub‐catchment boundaries (described in section 3.1 and Table 1), soil moisture stations (crosses, S01–S36), rain gauges (squares, N1–N4) and the piezometer (brown triangle, H09) used here

**FIGURE 3:**
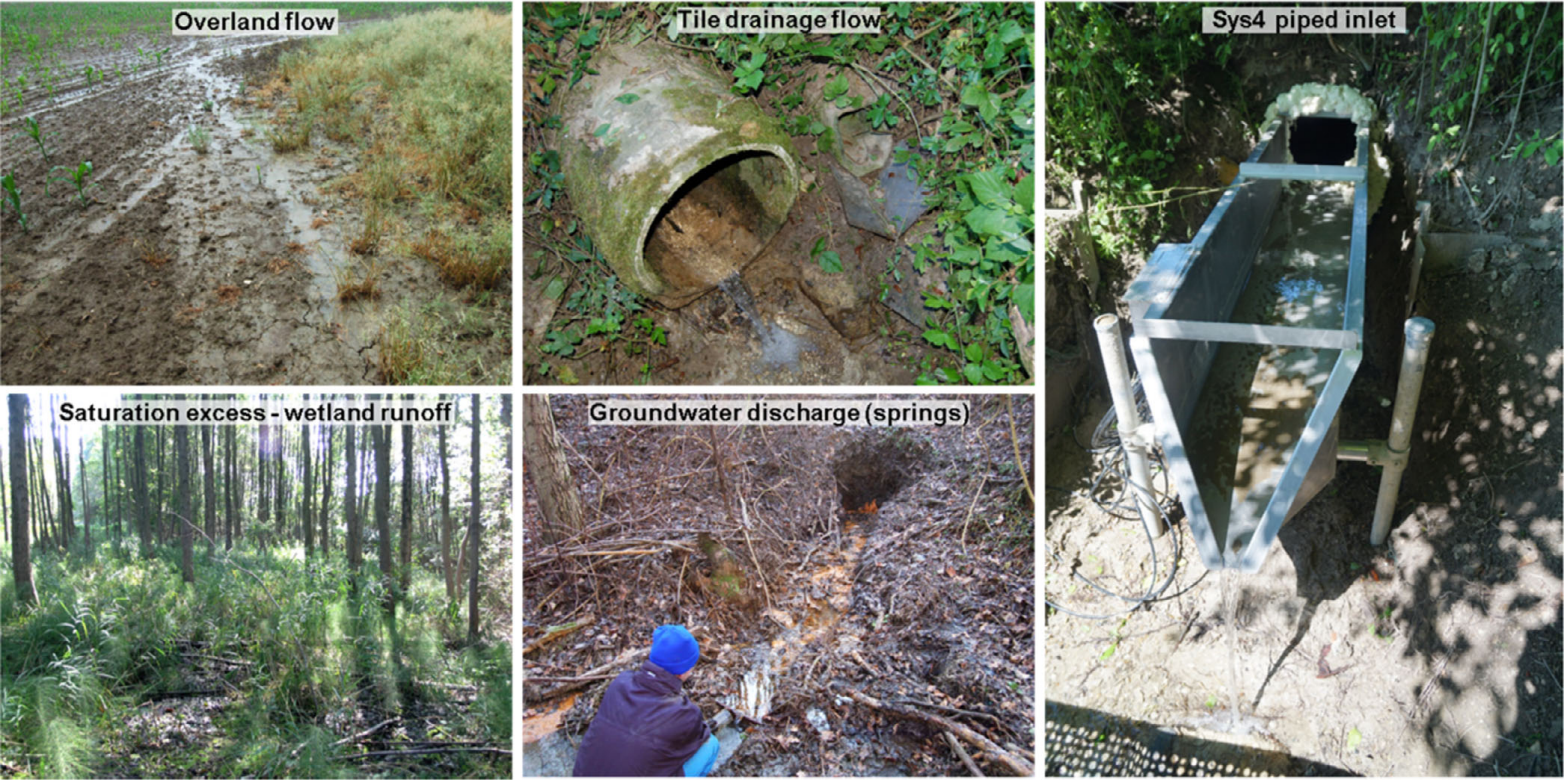
Runoff processes observed in the Hydrological Open Air Laboratory (HOAL) and piped inlet Sys4. Adjusted from Blöschl et al. (2016)

A range of runoff generation mechanisms are gauged in the HOAL (Figure 2, Figure 3), with the following characteristics during the study period (2014–2015): E1 and E2 are erosion gullies that represent overland flow from the agricultural fields entering the stream at several locations. Frau1 and Frau2 are tile drainage mechanisms that drain agricultural fields in the northwest of the catchment and have ephemeral flow. Sys2 and Sys3 are tile drainage mechanisms in the southeast and are perennial. These tile drainage mechanisms cover about 15% of the catchment and were installed in the 1950s to remedy waterlogging associated with the low‐permeability soils. A1 and A2 are perennial with quick event runoff from wetlands that seep into the stream via rivulets. Sys1 and Q1 are deep aquifer springs, identified by the runoff dynamics and chemical composition of runoff (Exner‐Kittridge et al., 2016). Sys4 represents flow from the former, most upstream part of the stream, which was piped in the 1940s, with contributions from two tile drainage mechanisms. Its dynamics and chemistry are similar to those of the perennial tile drainage mechanisms (Sys2 and Sys3). MW is the catchment outlet draining an area of 66 ha.

### 2.2 Hydro‐meteorological data

Discharge is monitored at 12 locations that have been selected to cover different runoff generation mechanisms (Table 1). Calibrated flumes measure discharge at a temporal resolution of 1 min. The drainage area of the flumes was delineated based on a digital elevation model (Lidar based data at 0.5 m spatial resolution, Figure 2). For A1, A2, Sys1 and Sys2 only the downslope drainage areas below the road running through the catchment were considered, as the road was often observed to act as a ridgeline disconnecting the upslope part of the sub‐catchments during events. The location of the road coincides with a clear change in topography, where west from the road the slopes are steeper. In the sub‐surface there is a lignite layer close to the surface west from the road, which acts as a natural ridgeline. Note that Sys3 has a drainage system that extends underneath the road.

**TABLE 1:** Sub‐catchments, associated soil moisture stations and type of runoff generation mechanism

Stream gauge	Associated soil moisture stations	Runoff dynamics during the study period	Runoff generation mechanisms
E1	6, 7, 8, 22, 21, 24, 25, 26, 28, 29, 30, 36	Ephemeral	Overland flow
E2	6, 7, 8, 22, 21, 24, 25, 26, 28, 29, 30, 36	Ephemeral	Overland flow
Frau1	28, 29	Ephemeral	Tile drainage
Frau2	24, 25, 26, 28	Ephemeral	Tile drainage
Sys2	14, 15	Perennial	Tile drainage
Sys3	15	Perennial	Tile drainage
A1	14, 15	Perennial	Wetland
A2	14	Perennial	Wetland
Sys1	13, 14	Perennial	Spring (deep aquifer)
Q1	29	Perennial	Spring (deep aquifer)
Sys4	1, 2, 4, 5, 6, 7, 8, 22, 36	Perennial	Piped inlet (aggregated mechanism)
MW	1, 2, 4, 5, 6, 7, 8, 10, 13, 14, 15, 16, 17, 21, 22, 24, 25, 26, 27, 28, 29, 30, 31, 32, 33, 34, 35, 36	Perennial	Catchment outlet (aggregated mechanism)

Four rain gauges measure precipitation at a temporal resolution of 1 min. Since the spatial variability of precipitation was small, the arithmetic mean of the four stations was used in the analysis.

Soil moisture measured at 0.05, 0.10 and 0.20 m depths at 32 stations is used, of which 21 are operated throughout the year, and 11 are temporary (removed during cultivation activities). The data gaps of the temporary stations (August to November) were filled by a linear regression with a donor permanent station each, identified based on similar temporal behaviour using the time stability approach of Vachaud et al. (1985). Correlation coefficients between donor permanent and temporary station ranged between *r* = 0.66 and 0.96 with one outlier of *r* = 0.54. Sub‐catchment average soil moisture was calculated with linear averaging over the stations located within each sub‐catchment or if there was none, the nearest station (Table 1). The vertical soil moisture profile was interpolated from the sensors at 0.05, 0.10, 0.20 m depths, and the average over the range 0.00–0.20 m was used in the analysis.

One piezometer (H09, Figure 2) located in the riparian forest close to the stream, was used as an indicator of groundwater levels. This piezometer has one of the most complete time series in the study period, and is located in the middle section of the stream, close to several streamflow gauges. Its groundwater dynamics are representative for piezometers in a transition between shallow riparian zone and steep hillslope locations as it is located on a lower slope according to the classification of Pavlin et al. (2021).

The study period is from January 2014 to December 2015, where the coverage of the soil moisture sensors was best. For further details on the instrumentation see Blöschl et al. (2016).

### 2.3 Event selection

To understand the controls on runoff peaks, we selected events from the runoff and precipitation records. Events were selected when all of these three criteria were satisfied:

- Hourly rainfall intensity exceeds 0.025 mm/hr, intended to separate zero and non‐zero precipitation.
- Total event rainfall exceeds 5 mm.
- The period between events without rainfall is at least 6 h.

**FIGURE 4:**
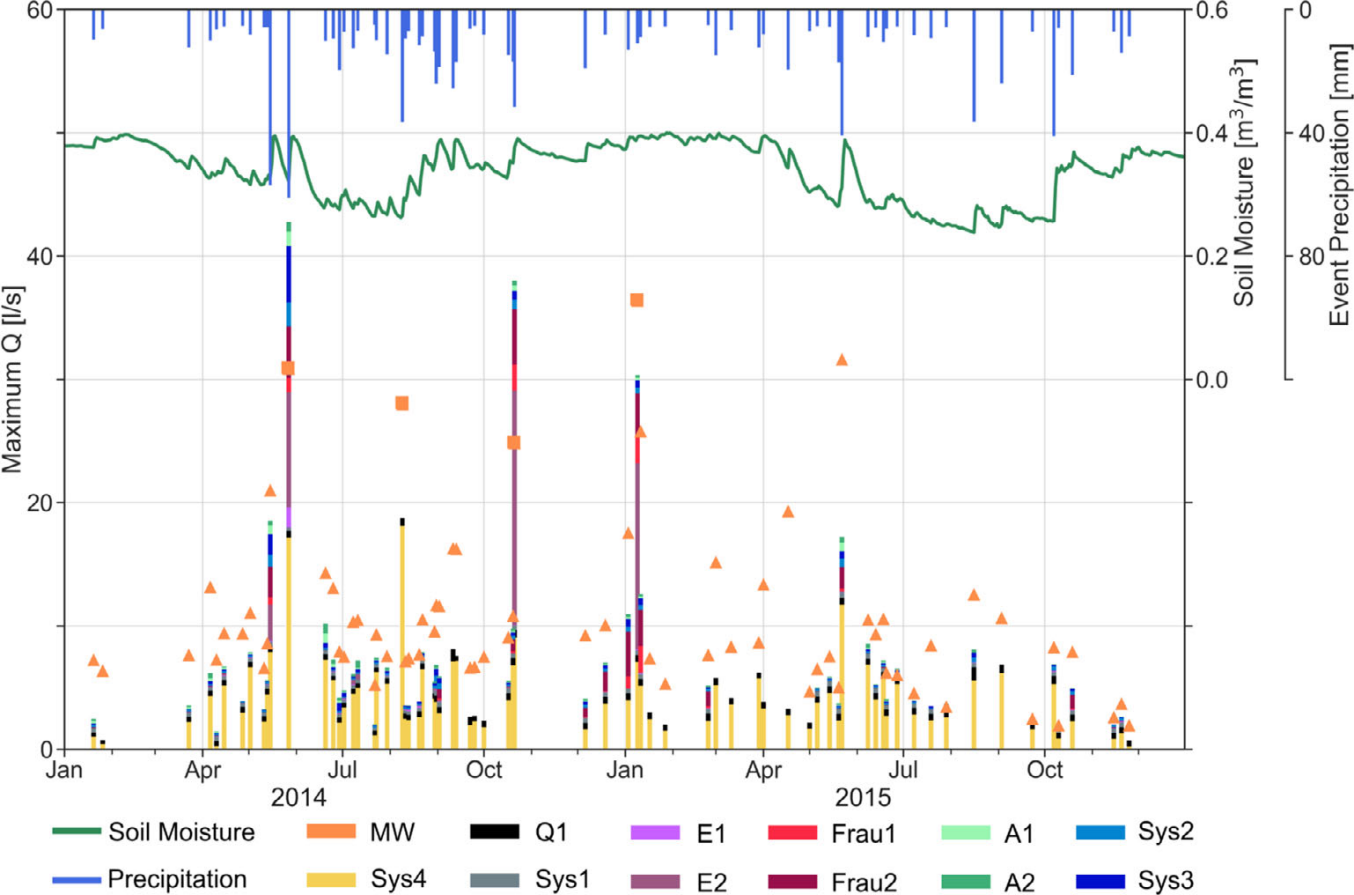
Overview of the 73 events used in this study during 2014 and 2015. Peak runoff at MW catchment outlet is shown as orange triangles. The four events discussed in section 4.3 are indicated as squares. Peak runoff of the sub‐catchments is indicated as stacked bars. Catchment average event precipitation (blue bars at the top) and catchment average soil moisture (0–0.20 m depth) (green line) are also shown

Figure 4 shows an overview of peak runoff, event precipitation and catchment average soil moisture of the events identified. Due to shifts in the timing and ungauged lateral inflow into the stream, peak runoff of the sub‐catchments does not add up to that at the catchment outlet. Out of 73 events, 20, 26, 17 and 10 occurred in spring, summer, autumn and winter, respectively.

### 2.4 Assessing the importance and non‐linearity of controls

For all events the hydrographs and soil moisture time series for each sub‐catchment were visually inspected to assess the quality of discharge, precipitation and soil moisture data. Furthermore, we evaluated if maximum soil moisture was reached during the event. The importance of event precipitation, maximum event soil moisture and mean event groundwater levels for event peak runoff was then evaluated by scatter plots and Spearman rank correlation coefficients.

**FIGURE A1:**
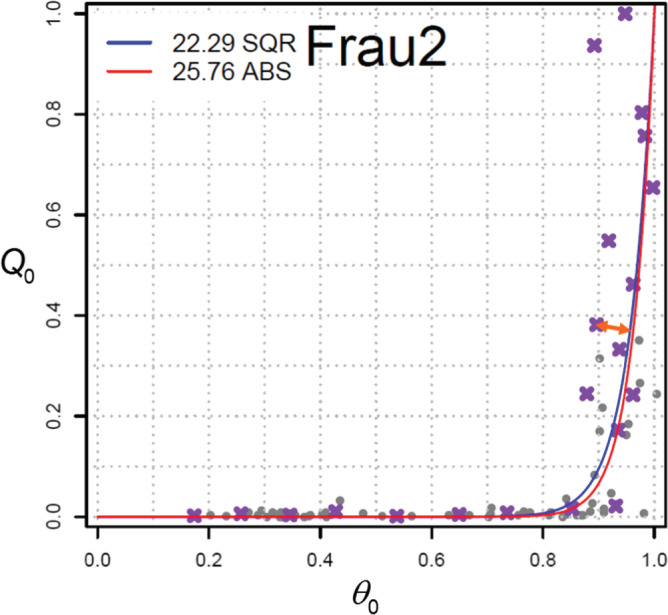
Illustration of the fitting of Equation (1). Green points are the original data, purple crosses are the data aggregated to a 0.1 × 0.1 grid, the blue is the fitted power laws using squared perpendicular distances between Equation (1) and the data points, and the orange arrow shows an example of the perpendicular distance. For comparison the fit with the alternative distance measure, absolute perpendicular distances, is shown in red

In a second step, we evaluated the non‐linearity in runoff generation. While non‐linearity can be defined in various ways, including polynomials, step functions, piecewise linear relationships and threshold behaviour (Choudhury et al., 2008; Rogger et al., 2012; Zehe & Blöschl, 2004), a common definition in runoff generation are power law relationships (Majone et al., 2010; McIntyre, 2013). Their advantages are that they are non‐dimensional and thus generalisable to other situations, and the exponent is a readily interpretable measure for the degree in non‐linearity. A preliminary analysis suggested that a power law gave generally better fits than other functions with the same number of parameters. We therefore fitted the following relationships for each stream gauge separately.

(1) $$Q_{0}=P_{0}^{a}\;Q_{0}=\theta_{0}^{b}\;Q_{0}=G_{0}^{c},$$

where $Q_{0}$ is the event peak runoff $P_{0},$ $\theta_{0},$ and $G_{0}$ are respectively event precipitation, maximum event soil moisture and mean event groundwater levels (all scaled by their maxima for each stream gauge separately), and *a*, *b*, *c* are parameters representing the degree of non‐linearity. The parameters were estimated by minimizing the sum of squared perpendicular distances between Equation (1) and the data points (see Figure A1). Since the data were rather unevenly distributed, we did not directly fit Equation (1) to the $Q_{0}$, $P_{0}$, $\theta_{0}$ and $G_{0}$ data. Instead we aggregated the data to a 0.1 × 0.1 grid in the space of, for example, $Q_{0}$ versus $P_{0}$ and thus obtained average $Q_{0}$ and $P_{0}$ values for each grid cell. In a second step we fitted Equation (1) to the $Q_{0}$ and $P_{0}$ averages of each grid cell, and proceeded in a similar way for $Q_{0}$ versus $\theta_{0}$ and $Q_{0}$ versus $G_{0}$. We tested the sensitivity of the parameter estimates to the objective function and the choice of a grid by repeating the fitting for an objective function involving absolute rather than squared distances and without a grid, and the differences were relatively minor.

## 3 RESULTS

### 3.1 Peak runoff response to precipitation, soil moisture and groundwater level

**FIGURE 5:**
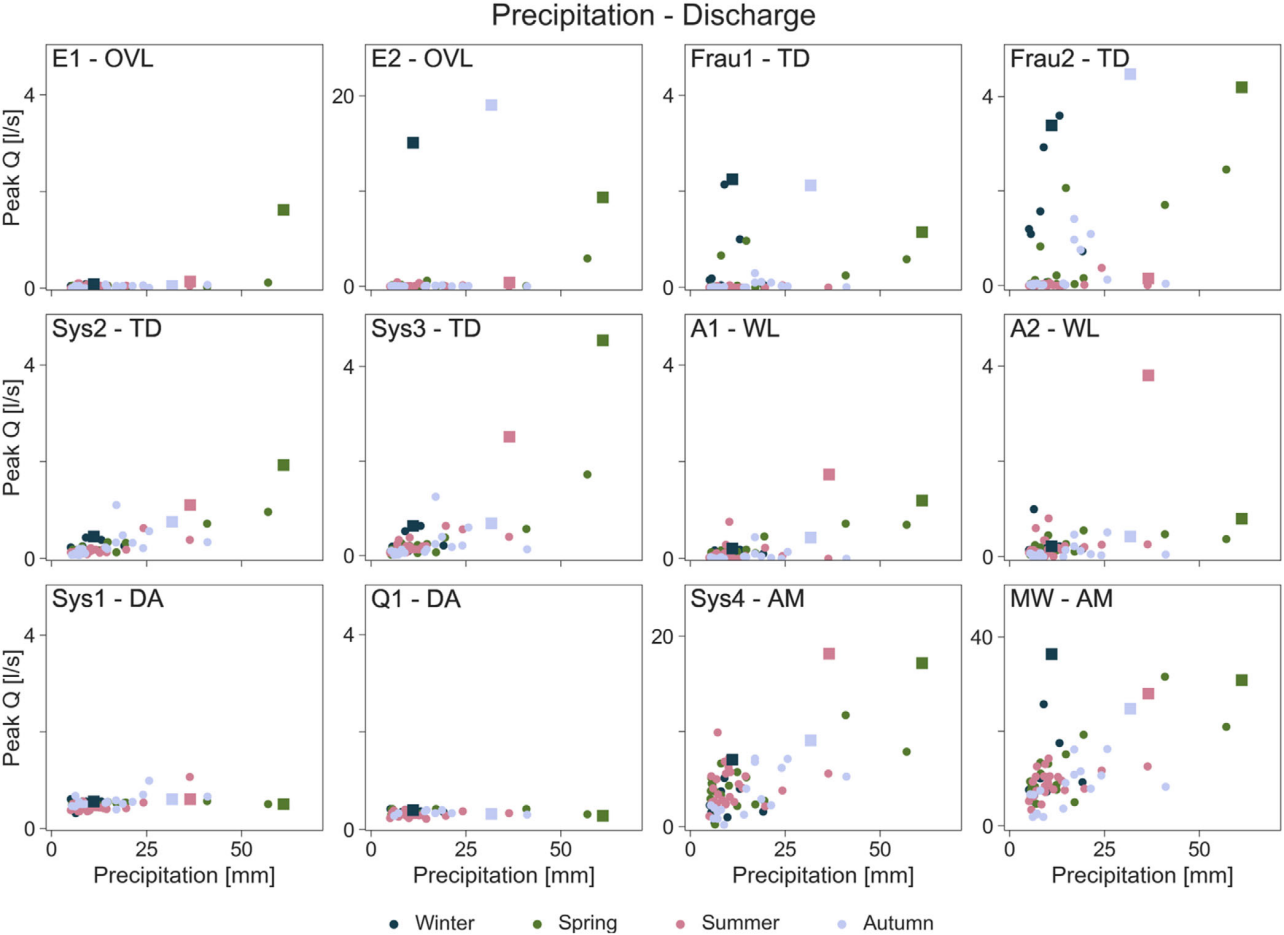
Relationship between event precipitation and peak runoff per sub‐catchment. Mechanisms (Table 1): Overland flow (OVL), tile drainage (TD), wetland (WL), deep aquifer (DA), aggregated mechanism (AM). Squares indicate the four events discussed in section 4.3. The seasons are defined as follows: winter (dark blue, December, January, February), spring (green, March, April, May), summer (pink, June, July, August) and autumn (grey, September, October, November)

There are apparent differences in the relationship of peak runoff and event precipitation between the runoff generation mechanisms (Figure 5). Runoff shows little relation to precipitation in the ephemeral overland flow (E1, E2) and tile drainage (Frau1, Frau2) mechanisms. For these mechanisms there is a clear distinction between seasons, where in winter (dark blue symbols) and to some degree spring (green symbols), smaller precipitation events lead to higher runoff than in the other seasons. In contrast, the relation is more pronounced in the perennial tile drainage mechanisms (Sys2, Sys3), but there is little difference between the seasons. The wetlands do not show a distinct relation to precipitation, although runoff in A2 responds more strongly to precipitation than A1. The deep aquifer mechanisms show no relation to precipitation, while for the aggregated mechanisms there is a strong positive relationship (Table 2).

**TABLE 2:** Spearman rank correlation coefficients *r* between peak runoff (*Q*), and event precipitation (*P*), soil moisture (*θ*) and groundwater level (*G*). The two highest correlations of each control are printed in bold. The *p* value is the probability that the null hypothesis (H_0_: Population correlation coefficient *ρ* = 0) is true. Column at the far right shows control with highest correlation. Mechanisms (Table 1): Overland flow (OVL), tile drainage (TD), wetland (WL), deep aquifer (DA), aggregated mechanism (AM)

Mechanism	Stream gauge	Precipitation		Soil moisture		Groundwater level		Max *r*
		*r*	*p*	*r*	*p*	*r*	*p*	
OVL	E1	0.58	0.00	0.39	0.03	0.15	0.41	P
OVL	E2	0.29	0.10	0.78	0.00	0.68	0.00	θ
TD	Frau1	0.44	0.01	**0.79**	0.00	0.59	0.00	θ
TD	Frau2	0.43	0.02	**0.81**	0.00	**0.72**	0.00	θ
TD	Sys2	**0.79**	0.00	0.68	0.00	0.42	0.02	P
TD	Sys3	**0.70**	0.00	0.60	0.00	0.28	0.14	P
WL	A1	0.37	0.04	0.79	0.00	**0.71**	0.00	θ
WL	A2	0.55	0.00	0.74	0.00	0.53	0.00	θ
DA	Sys1	0.42	0.02	0.19	0.29	−0.14	0.43	P
DA	Q1	−0.33	0.13	−0.01	0.97	0.26	0.24	G
AM	Sys4	0.52	0.00	0.24	0.18	0.25	0.18	P
AM	MW	0.57	0.00	0.58	0.00	0.46	0.01	θ

**FIGURE 6:**
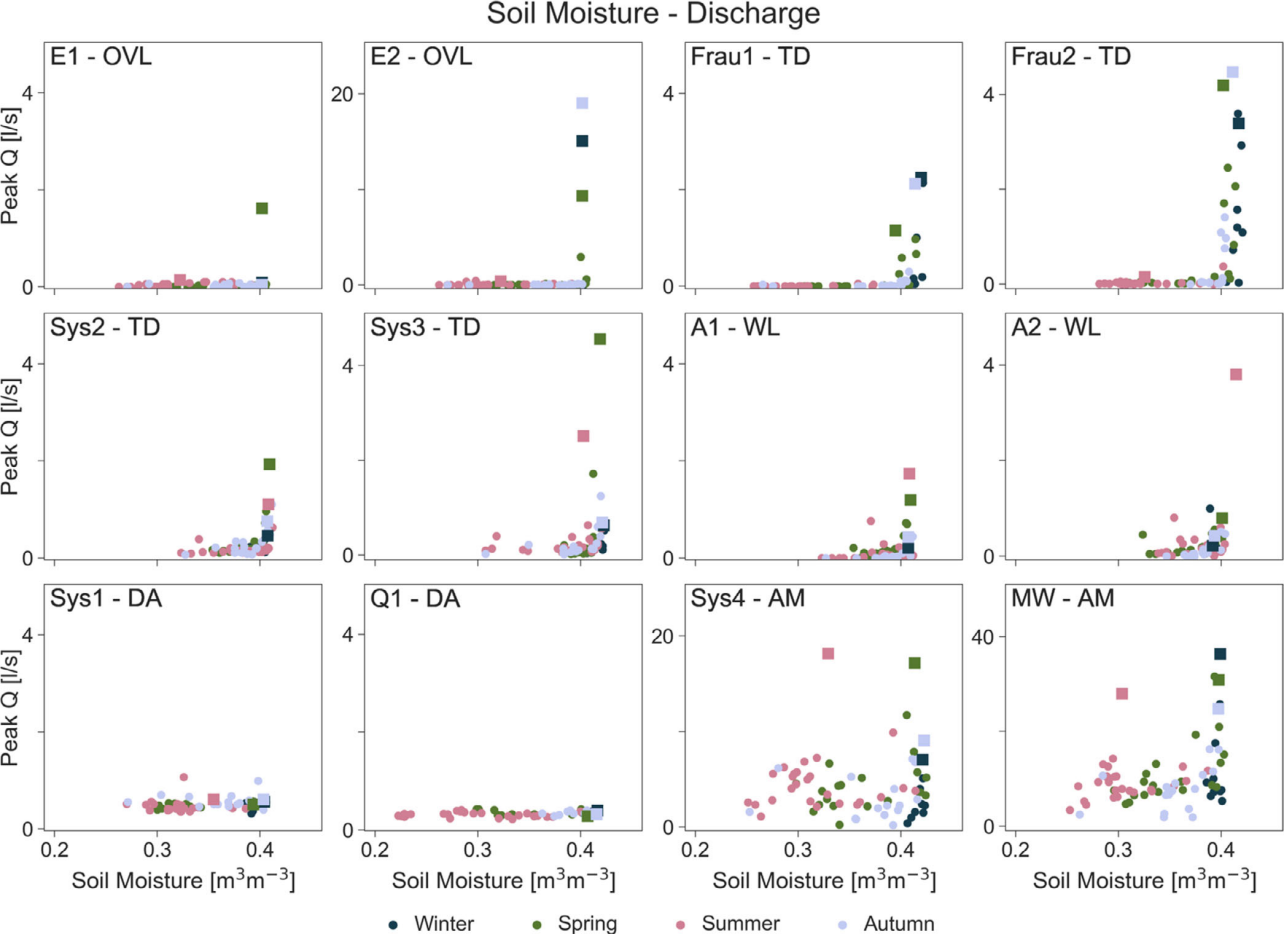
Relationship between maximum event soil moisture (0–20 cm depth) and peak runoff per sub‐catchment. Mechanisms (Table 1): Overland flow (OVL), tile drainage (TD), wetland (WL), deep aquifer (DA), aggregated mechanism (AM). Squares indicate the four events discussed in section 4.3

The differences between the runoff generation mechanisms are more pronounced for the runoff ‐ soil moisture relationship (Figure 6). In the overland flow and tile drainage mechanisms, runoff occurs only after a threshold is reached, with close to zero runoff below that threshold, and this relationship is consistent across seasons. The four tile drainage mechanisms exhibit a similar threshold behaviour for the large events but, below a threshold, some runoff occurs in Sys2 and Sys3, while there is no or almost no runoff at Frau1 and Frau2. In the wetland mechanisms, in particular A1, runoff increases more gradually with soil moisture. As expected, the deep aquifer mechanisms (Sys1, Q1) show no relation to soil moisture. For the aggregated mechanisms (Sys4, MW), the relationship is an aggregate of those of the different mechanisms within the catchment.

**FIGURE 7:**
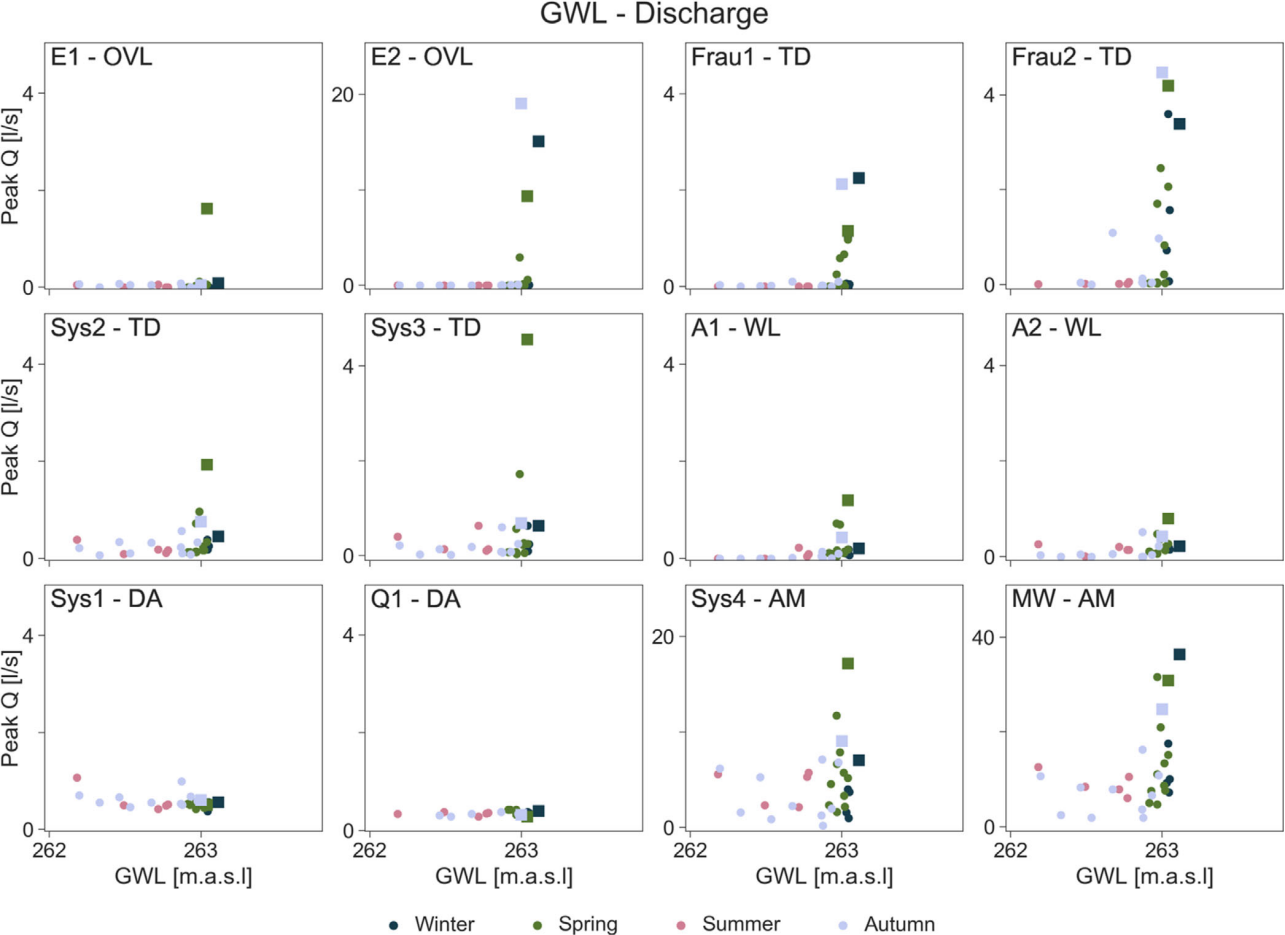
Relationship between mean event groundwater level and peak runoff per sub‐catchment. Mechanisms (Table 1): Overland flow (OVL), tile drainage (TD), wetland (WL), deep aquifer (DA), aggregated mechanism (AM). Squares indicate the four events discussed in section 4.3

The relationships of runoff and groundwater level are similar to soil moisture (Figure 7). A clear threshold behaviour can be observed in the overland flow and tile drainage mechanisms. This threshold is less pronounced in the wetlands. No relation is observed between runoff peak and groundwater level in the deep aquifer mechanisms, because these mechanisms contribute more to baseflow than to event runoff (Pavlin et al., 2021). While the aggregated mechanisms show a certain threshold behaviour as these mechanisms aggregate contributions from overland flow, tile drain and wetland mechanisms besides the deep aquifer contributions.

### 3.2 Relative importance of controls on peak runoff and non‐linearity

The relative importance of precipitation, soil moisture and groundwater level for peak runoff is quantified by the Spearman rank correlation coefficient *r* (Table 2). Note that usually maximum soil moisture was reached during the event in every sub‐catchment. For the overland flow mechanism E2, runoff peaks are significantly correlated with soil moisture and groundwater level, while for E1 the correlations with precipitation are stronger. The ephemeral tile drainage mechanisms (Frau1, Frau2) are most highly correlated with soil moisture (*r* around 0.8). In contrast, the perennial tile drainage mechanisms (Sys2, Sys3) are most highly correlated with precipitation (*r* = 0.79 and 0.70, respectively) and to a lesser degree with soil moisture (*r* = 0.68 and 0.60). The stronger control of precipitation on the perennial tile drainage mechanisms, as compared to the ephemeral tile drainage mechanisms, is likely related to a more continuous connection of the flow paths to the stream. In the ephemeral tile drainage mechanisms, a given precipitation depth may or may not produce runoff, depending on the antecedent soil moisture state. The runoff peaks of the wetland mechanisms (A1, A2) are closely related to soil moisture (*r* = 0.79, 0.74) and groundwater level (*r* = 0.71, 0.53), and to a lesser extent to precipitation (*r* = 0.37, 0.55). The smaller role of precipitation is likely because of the significant seasonal dynamics of soil moisture and groundwater in these mechanisms. The deep aquifer mechanisms (Sys1, Q1) show low correlations to groundwater (*r* = −0.14, 0.26) and precipitation (*r* = 0.42, −0.33), and less correlation with soil moisture (*r* = 0.19, −0.01). The low correlations suggest that the flow paths of these mechanisms are disconnected from the unsaturated soil at the event scale.

The aggregated mechanisms (Sys4, MW) show a significant correlation with precipitation (*r* = 0.52 and 0.57), with MW exhibiting an almost equally strong correlation with soil moisture (*r* = 0.58). This stronger dependence on soil moisture in MW, compared to Sys4, reflects the strong dependence of the overland flow and tile drainage mechanisms to soil moisture (*r =* 0.39–0.81) and their contribution to the catchment outlet.

The Spearman rank correlation assesses the association of peak runoff with the controls irrespective of the shape of this relationship. To quantify the degree of non‐linearity between peak runoff and each of the controls, we fitted Equation (1). The results are shown in Table 3. Consistent with the visual appearance in Figures 5, 6, 7, the overland flow mechanisms (E1, E2) show the largest degree of non‐linearity with soil moisture on the order of *b* = 80, which is essentially threshold behaviour close to its maximum. With this parameter, *Q*_*0*_ = 0.02 and 0.45 for $\theta_{0}$ = 0.95 and 0.99, respectively. The degree of non‐linearity is smaller for the relationship with groundwater level and precipitation. The tile drainage mechanisms (Frau1, Frau2, Sys2, Sys3) are also highly non‐linear for soil moisture (*b* around 20; so *Q*_*0*_ = 0.36 and 0.82 for $\theta_{0}$ = 0.95 and 0.99, respectively) with slightly lower values of *b* for the perennial (Sys2, Sys3) than for the ephemeral (Frau1, Frau2) mechanisms, because of the larger groundwater contribution. The non‐linearity for groundwater levels is similar, but that to precipitation is much lower (between 1.1 and 6.1) because of the dominance of sub‐surface controls. In the wetlands (A1, A2), soil moisture and groundwater exhibit more non‐linear controls than precipitation, although the degree of non‐linearity is smaller than that of the tile drainage mechanisms. The lower non‐linearity of A2 than A1 is expected because A2 is believed to be better connected to the groundwater than A1, due to its higher baseflow and larger diurnal streamflow fluctuations in response to evaporation (Széles et al., 2018).

**TABLE 3:** Degree of non‐linearity between peak runoff (*Q_0_*), and event precipitation (*P_0_*), soil moisture ($\theta_{0}$) and groundwater level (*G*_*0*_) (all scaled) (Equation (1))

		Non‐linearity parameters *a, b, c*		
Mechanism	Stream gauge	*a* precipitation	*b* soil moisture	*c* groundwater level
OVL	E1	38.2	85.4	62.4
OVL	E2	4.4	79.5	24.5
TD	Frau1	6.1	20.7	17.5
TD	Frau2	1.1	22.3	16.3
TD	Sys2	1.7	17.1	12.8
TD	Sys3	3.3	18.8	21.0
WL	A1	2.2	18.5	13.5
WL	A2	0.9	8.4	7.3
DA	Sys1	1.0	3.6	1.4
DA	Q1	0.2	1.1	0.3
AM	Sys4	1.2	5.4	8.2
AM	MW	0.9	6.4	5.4

The deep aquifer mechanisms (Sys1, Q1) are not significantly correlated with precipitation, soil moisture or groundwater level (except for Sys1 to precipitation) at the scale of individual events (Table 2) so, while values of *a*, *b*, *c* around one imply almost linear relationships, they contain a lot of scatter (Figures 5, 6, 7).

Finally, the aggregated mechanisms (Sys4, MW) give non‐linearity parameters of around six for soil moisture and groundwater levels, which are between those of the ephemeral mechanisms and those of the deep aquifer mechanisms. This is expected because of aggregation effects in the catchment. At the MW (66 ha) scale, soil moisture and groundwater level show some non‐linear behaviour. In contrast, the threshold behaviour visible for the overland mechanisms is lost for precipitation.

### 3.3 Seasonal behaviour of runoff hydrographs

**FIGURE 8:**
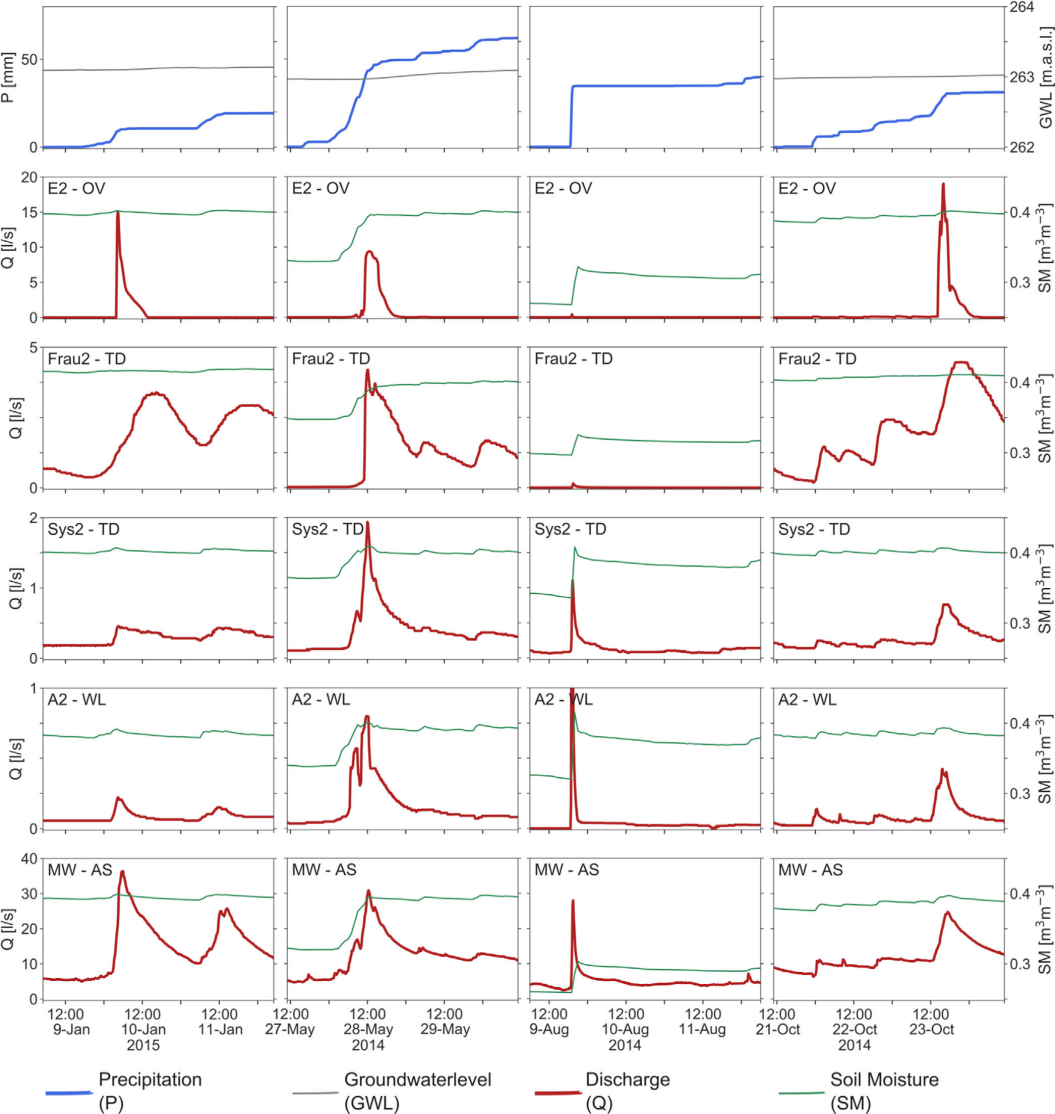
Hydrographs of events in winter, spring, summer and autumn (left to right) for five sub‐catchments with different runoff generation mechanisms. All hydrographs cover 3 days around the onset of the event. Top panels: Cumulative precipitation (blue) and groundwater level (grey). Remaining panels show runoff (red) and soil moisture (green). Top to bottom: Overland flow (OV), tile drainage ephemeral (TD), tile drainage perennial (TD), wetland (WL) and aggregated mechanism (AM)

To assist in the interpretation of the above analyses of peaks concerning their controls and season, Figure 8 shows typical examples of storm hydrographs in winter, spring, summer and autumn.

- Winter event (January 2015): January 2015 was a wet month, and there was snow on the ground, which melted and further increased soil moisture close to saturation in all sub‐catchments. The relatively small rainfall event (20 mm) therefore led to unusually high runoff in the overland flow and tile drainage mechanisms. While the overland flow mechanism showed a short lag time until activation of the mechanism, the tile drainage mechanism was already activated and showed a slow rise in runoff. In the wetland mechanism, the runoff was not particularly high because of the small rainfall depth. At the catchment outlet, the event peak in January 2015 was the highest in the 2 year study period, with a large contribution from the overland flow and tile drainage mechanisms.
- Spring event (May 2014): Antecedent soil moisture of this event was lower than for the winter event, leaving room for infiltration at the beginning of the event. The event rainfall was higher (about 50 mm). In both the overland flow and tile drainage mechanisms, runoff commenced once soil moisture had built up, so runoff response was delayed. Because of the surface flow paths (Silasari et al., 2017), the recession of the overland flow was steep, while the tile drainage mechanism stayed activated for longer. In the wetland mechanism, the runoff response had very little delay and there was a direct correspondence of rainfall time patterns with those of runoff with two short runoff peaks resulting from two short rainfall peaks. The rise in runoff at the catchment outlet started simultaneously with the wetlands, and a small drop in runoff could be observed after the first rainfall peak. The peak was concurrent with those of the overland flow and tile drainage mechanisms. There was sub‐stantial baseflow after the event, corresponding to that of the tile drainage mechanisms, with additional contributions from the deep aquifer.
- Summer event (August 2014): Antecedent soil moisture of the summer event was much lower than that in winter and spring. 35 mm of event precipitation in 1 hour increased soil moisture sharply, but saturation was not reached. The overland (E2) and ephemeral tile drainage (Frau2) mechanisms showed no runoff response. The perennial tile drainage mechanism (Sys2) showed a very short response. Similarly, the response of the wetland mechanism (A2) was very flashy and the runoff peak was higher than for the other events, indicating that the flow paths during this event were superficial or very shallow, in line with the short duration of the storm. At the catchment outlet, the response was similar to those of the flashy sub‐catchments, and baseflow after the event was rather low. The comparison with the winter event is striking. The overland flow mechanism E2 had a strong response in winter, but no response in summer; the wetland mechanism A2 had almost no response in winter and a strong response in summer.
- Autumn event (October 2014): In October antecedent soil moisture was high. The rainfall was low intensity, long duration. The overland mechanism had a long delay and only responded when rainfall intensities were higher at the end of the event. In contrast, the tile drainage mechanism was already activated and responded immediately to precipitation. The buildup of runoff in the ephemeral tile drainage mechanism (Frau2) was more pronounced than in the perennial mechanism (Sys2). In the wetland mechanism there was an immediate but modest response to precipitation in line with the low‐precipitation intensities. At the catchment outlet, the contribution of the tile drainage and wetland mechanisms played an important role at the beginning of the event, while the central peak of 25 L/s was mainly due to the overland flow mechanism (peak flow at E2 of 20 L/s).

## 4 DISCUSSION

### 4.1 Seasonality and role of controls on runoff generation

During winter, small rainfall events tend to lead to high runoff in the ephemeral overland flow and tile drainage mechanisms, and thus at the catchment outlet which, along with the immediate response to rainfall, indicates persistent connectivity in the sub‐surface (Szeles et al., 2018). In the other sub‐catchments, where soil moisture is somewhat lower and thus some storage volume is available, the highest runoff occurs with the largest event precipitation. During spring and autumn, runoff from the ephemeral tile drainage and overland flow mechanisms starts when soil moisture is close to soil saturation (about 95% of its observed maximum). There is thus a switching behaviour, also observed in other studies (Grayson et al., 1997; Penna et al., 2011, 2015; Ross et al., 2021), from no runoff to significant runoff. During summer, soil moisture and groundwater levels tend to be low for all sub‐catchments (Figures 5 and 6), and even with large rainfall events no runoff is observed from the ephemeral mechanisms. In the other mechanisms, the response tends to be flashy.

The seasonal contrast in soil moisture and the associated runoff generation contribute to establishing the relative importance of the individual controls on peak runoff. Table 2 suggests that, for the ephemeral tile drainage mechanisms, soil moisture is the main control (although both event precipitation and groundwater level are also important), which is aligned with the lower runoff in summer and the larger runoff in winter. The wetland mechanisms are similar in terms of their main controls and this may be related to the sub‐catchment areas partly drying out in summer, so variations in antecedent soil moisture are important for runoff generation. In addition, the wetlands are strongly affected by variations in groundwater level. Since the water table is close to the surface it may affect the size of the saturation areas and thus runoff. On the other hand, the perennial tile drainage mechanisms are more sensitive to precipitation (Table 2) and events vary less between seasons, likely because flow paths are deeper, and soil moisture may be less relevant.

Seasonal differences in threshold behaviour have in particular been found in previous studies in forested catchments. For example, data from three small forested watersheds in North Carolina, USA (Scaife & Band, 2017) showed that, during the growing season, when water stress is high, runoff generation thresholds as a function of antecedent soil moisture and precipitation tend to be lower than during the dormant season for the same soil moisture conditions, which the authors interpreted as being a result of the forest canopy response to water stress. Similarly, data from a 3 km^2^ forested catchments in Southern China (Wei et al., 2020) indicated that, during the growing and rainy seasons, canopy interception resulted in greater sensitivity of runoff to precipitation than in the dormant season. Also runoff generation thresholds were lower, probably due to higher soil moisture in areas close to the stream. While the forested area in the HOAL is much less than in the above studies, seasonality seems to be equally important and similar to the study of Saffarpour et al. (2016). In their 1 ha grassland catchment in South‐East Australia, the strong soil moisture seasonality resulted in clear changes in runoff response and the associated runoff generation mechanisms. In autumn, at the beginning of the runoff season, saturation excess overland flow in the riparian zone was dominant. As the season progressed, soil water storage across the catchment increased and the entire hillslopes connected to the riparian zone and contributed to streamflow. These seasonal changes in the connectivity can also be found in the HOAL (Széles et al., 2018).

### 4.2 Non‐linearity of runoff generation

The scatter plots (Figures 5, 6, 7) and the statistical analysis (Table 3) showed very clear differences in the degree of non‐linearity of runoff generation between the different mechanisms. The most non‐linear mechanisms are the overland flow mechanisms with non‐linearity parameters for soil moisture of around *b* = 80. This high parameter value implies that there is essentially no runoff below a threshold, and above that threshold runoff increases abruptly. This has also been demonstrated by the work of Tiefenbacher et al. (2021), who identified clear thresholds for the switch between surface and subsurface runoff generation for their rainfall simulation experiments with a HOAL cambisol. Given that both rainfall and soil moisture are important and highly non‐linear controls (Tables 2, 3), it is likely that this threshold behaviour is related to both infiltration excess and saturation excess runoff generation. During winter, spring and autumn, when the largest events occur, the surface tends to saturate when the perched groundwater table intersects with the surface (Silasari et al., 2017) and surface flow paths connect (Gomi et al., 2008), so the infiltration excess mechanism prevails. On the other hand, given observed rainfall intensities, it is also possible that infiltration excess occurs occasionally and/or locally. As noted by Szilagyi (2007), non‐linear runoff response may be due to both surface and sub‐surface processes, as higher rainfall intensity produces surface runoff sooner through infiltration excess and brings the aquifer closer to saturation in a shorter time, leading to faster response.

The ephemeral tile drainage mechanisms show non‐linearity parameters for soil moisture of around *b* = 20. In this case, the threshold behaviour is likely related to the onset of preferential flow in the soil profile. The drainage tiles are about 70 cm below ground, and the fast response indicates that preferential vertical flow is very important. A switch from matrix flow to preferential flow may induce a very non‐linear threshold relationship at the plot scale (Zehe et al., 2010; Zehe & Blöschl, 2004). The groundwater level is also relevant, and in some areas, the threshold may also be related to the moment when an intersection of the groundwater table with the drain pipes occurs.

The perennial tile drainage mechanisms are associated with deeper flow paths, so there is more access to groundwater storage, which reduces the non‐linear behaviour of the response. Non‐linearity of runoff generation occurs mainly for soil moisture and groundwater level, so both the onset of macropore flow and the intersection of the groundwater table with the drain pipes can be relevant mechanisms. Additionally, the direction of sub‐surface flow may change between the seasons (Bonanno et al., 2021) which will contribute additional complexity to the flow system and perhaps contribute to the degree of non‐linearity.

Even though the shallow depth to the groundwater table suggests that the wetlands have shallow flow paths, their degree of non‐linearity is similar to that of the perennial tile drainage mechanisms. This may be partly due to the seasonality of soil moisture, and partly to a functional similarity between the intersection of the groundwater table with the tile drains (in the case of Sys2, Sys3) and the intersection of the groundwater table with the ground surface (in the case of A1, A2). Once a soil moisture threshold is exceeded, much of the hillslopes may contribute to runoff in addition to the riparian zone (Detty & McGuire, 2010; Meyles et al., 2003; Penna et al., 2011; Sidle et al., 1995, 2000). The relative spatial distribution of the saturation areas and their dynamics then determine the degree of non‐linearity.

Event response of the deep aquifer mechanisms is not at all related to soil moisture, groundwater level or precipitation, and this is expected due to the deep flow paths. These mechanisms do show some seasonality, with higher runoff in winter and spring when soils are wetter, but the lag times are such that no consistent patterns emerge. Pavlin et al. (2021) showed that groundwater connectivity to the stream on the seasonal scale is higher than that on the event scale in the HOAL, indicating that groundwater contributes more to baseflow than to event runoff, which is consistent with the low correlations found here.

### 4.3 Scaling to the catchment scale and implications for modelling

The nested nature of the flow observations in the present study allows an analysis of how the individual hillslopes contribute to the runoff at the outlet of the entire catchment, both in terms of the event peak flow magnitudes as well as in terms of the degree of non‐linearity. The data suggest that the contributions of sub‐catchments depend both on the runoff generation mechanism and the season. Runoff from the aggregated mechanism Sys4 is mainly controlled by precipitation, which is due to the contribution of a shallow aquifer to Sys4. Hence, runoff in Sys4 even occurs when the soils are dry in summer (Figures 5 and 6), and the non‐linearity is rather low, similar to that of perennial tile drainage mechanisms. This is expected as two tile drainage mechanisms contribute to runoff at Sys4.

For the aggregated mechanism MW, the individual hillslope contributions can be more explicitly accounted for. In winter and spring, the hillslopes with the shallowest flow paths (overland flow and ephemeral tile drainage mechanisms) may have very significant runoff response, because of the high‐soil moisture, while the response of the wetland mechanisms is more sub‐dued, because of the relatively low‐rainfall intensities. This means that the largest winter and spring events observed at the catchment outlet are due to contributions from hillslopes with shallow flow paths. On the other hand, in summer, the hillslopes with the shallowest flow paths usually do not exceed the soil moisture threshold needed for runoff generation, while the wetland mechanisms, and to some degree the perennial tile drainage mechanisms, provide very significant response because of the high‐rainfall intensities. Therefore, the largest summer events observed at the catchment outlet are due to contributions from other hillslopes, those where flow paths are deeper or where part of the hillslope is saturated throughout the year. These differences highlight the need for understanding the spatial variability of soil moisture for predicting catchment response (Kim et al., 2016).

The aggregation from the hillslopes to the catchment also sheds light on the role of scale in non‐linear runoff generation. One generally expects that the runoff response becomes more linear with increasing scale because of aggregation effects (Sivapalan, 2003) because linearly aggregated random variables (following the central limit theorem) tend towards the normal distribution consistent with linear systems behaviour (Blöschl & Zehe, 2005). The findings of this study only partly support this notion. Runoff response at the catchment outlet is a mixture of the responses from the hillslopes, so the aggregate non‐linearity is also a mixture of those of the contributing hillslopes. Some of the threshold behaviour of the ephemeral mechanisms at the hillslope scale is not averaged out at the catchment scale, because these hillslopes dominate the catchment behaviour during some winter events. This is in line, for example, with the dominance of the sub‐catchment with largest storage potential in dominating the response of a Canadian prairie catchment (Spence, 2007). On the other hand, there is a tendency for the non‐linearity to decrease with catchment scale, which is related to the larger sub‐surface contribution to the events as the catchment scale increases. The contributions of the deep aquifer mechanisms and the diffuse groundwater contributions along the stream reduce the non‐linearity as one moves from the hillslope to the catchment scale, because during much of the year, the stream in the HOAL is gaining (Exner‐Kittridge et al., 2016; Eder et al., 2021). The more important role of sub‐surface flow paths with increasing catchment scale may thus be more relevant to a decreased non‐linearity than the aggregation per se.

The findings of this study have implications for hydrological modelling. If a catchment shows strongly non‐linear behaviour, small inaccuracies in precipitation and soil moisture will amplify, which will increase the uncertainty of hydrological forecasts relative to precipitation (Komma et al., 2007). Information on the degree of non‐linearity of runoff generation may inform the choice of rainfall‐runoff model parameters, for instance the *β* non‐linearity parameter in the relationship between runoff generated and soil moisture of HBV type models (Bergström, 1995), which is similar to the *b* parameter of this study. Based on calibration to streamflow series in 308 catchments in Austria, Merz and Blöschl (2004) found larger *β* values in the more arid parts of Austria, which is consistent with the propensity for convective events in these regions that are prone to infiltration excess runoff (Breinl et al., 2020). On the other hand, the moderate reduction of the degree of non‐linearity with catchment scale found here (unless there is a strong baseflow contribution) may explain why the calibrated *β* parameters of the study of Merz et al. (2009) did not decrease with catchment scale, although their catchments were much larger than the ones examined here. The non‐linear behaviour may also affect flood frequencies. For example, the decrease in the coefficient of variation (CV) of flood frequency distributions with catchment area has been explained (to some degree) by the increasing baseflow contribution to event runoff (e.g., Blöschl & Sivapalan, 1997), which is in line with a lower non‐linearity in larger catchments. As observed in the ephemeral mechanisms here, a high non‐linearity may also contribute to step changes in the flood frequency curves (Rogger et al., 2012; Viglione et al., 2009).

## 5 CONCLUSIONS AND OUTLOOK

An analysis of runoff events observed at 12 flumes representing small nested catchments or hillslopes with different runoff generation mechanisms allows us to draw the following conclusions:

- The relative importance of the controls on event peak runoff depends on the runoff generation mechanism.
- In the ephemeral tile drainage mechanisms, soil moisture is a more important control for runoff generation than precipitation because of the much larger peak runoff in winter when the soil is wet than during summer when it is dry. In the perennial tile drainage mechanisms, precipitation is more important for runoff generation because of the deeper flow paths that make runoff generation less sensitive to soil moisture changes in the top 20 cm analysed here. In the wetland mechanisms, soil moisture is the most important control because of seasonal variations in the size of the saturation areas.
- The degree of non‐linearity of the relationship between event peak runoff and its controls (soil moisture, event precipitation, groundwater level) depends on the runoff generation mechanisms.
- Ephemeral overland flow mechanisms are the most non‐linear. Runoff starts at a threshold of about 95% of the maximum soil moisture. Tile drainage and wetland mechanisms are less non‐linear because of deeper flow paths and the availability of permanently stored water in the system.
- The largest winter and spring events observed at the catchment outlet are due to contributions from hillslopes with shallow flow paths (ephemeral overland flow and tile drainage mechanisms), while the largest summer events are due to contributions from other hillslopes, those where flow paths are deeper or where part of the hillslope is saturated throughout the year (perennial tile drainage mechanisms and wetlands).
- Runoff response at the catchment outlet is a mixture of those of the runoff generation mechanisms it contains, so the aggregate non‐linearity is also a mixture of those of the contributing hillslopes. Sub‐surface contributions to event runoff, both from tributaries as well as from diffuse inflows along the stream, tend to reduce the non‐linearity as one moves from the hillslope to the catchment scale.
- Understanding the different degrees of non‐linearity of the runoff generation mechanisms may assist in the choice of parameters of rainfall‐runoff models and the shape of the flood frequency curve.

Soil moisture measurements with high spatial and temporal resolutions, along with runoff data at similar resolutions, provide an opportunity for understanding how the point and hillslope scale processes influence the entire catchment behaviour. As the catchment scale increases, in situ soil moisture measurements become less feasible and remote sensing observations gain importance. With the launch of high‐resolution synthetic aperture radars by the European Space Agency, the Copernicus Sentinel‐1 missions, it is now possible to obtain soil moisture estimates at a 1 km resolution every 2–3 days. Future work could therefore focus on extending the research of this paper by analysing such remote sensing observations along with runoff observations at larger catchment scales.

## Data Availability

The data that support the findings of this study are available from the corresponding author uponreasonable request.
